# Clinical Nomogram for Predicting Survival Outcomes in Early Mucinous Breast Cancer

**DOI:** 10.1371/journal.pone.0164921

**Published:** 2016-10-19

**Authors:** Jianfei Fu, Lunpo Wu, Mengjie Jiang, Dan Li, Ting Jiang, Zhongwu Hong, Fan Wang, Shuguang Li

**Affiliations:** 1 Department of Oncology, Zhejiang University Jinhua hospital, Jinhua, Zhejiang Province, China; 2 Department of Gastroenterology, Second Affiliated Hospital of Zhejiang University School of Medicine, Hangzhou, Zhejiang Province, China; 3 Institute of Gastroenterology, Zhejiang University, Hangzhou, Zhejiang Province, China; 4 Department of Radiation Oncology, The First Affiliated Hospital of ZheJiang Chinese Medical University, Zhejiang Provincial Hospital of TCM, Hangzhou, Zhejiang Province, China; 5 Department of Medical Oncology, Second Affiliated Hospital of Zhejiang University School of Medicine, Hangzhou, Zhejiang Province, China; 6 Cancer Institute (Key Laboratory of Cancer Prevention and Intervention, Chinese National Ministry of Education; Key Laboratory of Molecular Biology in Medical Sciences, Zhejiang Province, China), Second Affiliated Hospital of Zhejiang University School of Medicine, Hangzhou, Zhejiang Province, China; 7 Department of Nuclear Medicine, Zhejiang University Jinhua hospital, Jinhua, Zhejiang Province, China; University of North Carolina at Chapel Hill School of Medicine, UNITED STATES

## Abstract

**Background:**

The features related to the prognosis of patients with mucinous breast cancer (MBC) remain controversial. We aimed to explore the prognostic factors of MBC and develop a nomogram for predicting survival outcomes.

**Methods:**

The Surveillance, Epidemiology, and End Results (SEER) database was searched to identify 139611 women with resectable breast cancer from 1990 to 2007. Survival curves were generated using Kaplan-Meier methods. The 5-year and 10-year cancer-specific survival (CSS) rates were calculated using the Life-Table method. Based on Cox models, a nomogram was constructed to predict the probabilities of CSS for an individual patient. The competing risk regression model was used to analyse the specific survival of patients with MBC.

**Results:**

There were 136569 (97.82%) infiltrative ductal cancer (IDC) patients and 3042 (2.18%) MBC patients. Patients with MBC had less lymph node involvement, a higher frequency of well-differentiated lesions, and more estrogen receptor (ER)-positive tumors. Patients with MBC had significantly higher 5 and10-year CSS rates (98.23 and 96.03%, respectively) than patients with IDC (91.44 and 85.48%, respectively). Univariate and multivariate analyses showed that MBC was an independent factor for better prognosis. As for patients with MBC, the event of death caused by another disease exceeded the event of death caused by breast cancer. A competing risk regression model further showed that lymph node involvement, poorly differentiated grade and advanced T-classification were independent factors of poor prognosis in patients with MBC. The Nomogram can accurately predict CSS with a high C-index (0.816). Risk scores developed from the nomogram can more accurately predict the prognosis of patients with MBC (C-index = 0.789) than the traditional TNM system (C-index = 0.704, *P*< 0.001).

**Conclusions:**

Patients with MBC have a better prognosis than patients with IDC. Nomograms could help clinicians make more informed decisions in clinical practice. The competing risk regression model, as a more rational model, is recommended for use in the survival analysis of patients with MBC in the future.

## Introduction

Breast cancer is the most common cancer in women worldwide. Mucinous breast cancer (MBC) is a rare and special type, presenting with substantial extracellular mucin, and its incidence was reported to range from 1% to 6% for all primary breast cancers [1–4]. MBC is distinct from breast cancer, and its uniqueness should be considered in clinical practice. MBC is commonly seen in elderly, postmenopausal patients and is generally considered to have a favorable prognosis. Previous studies have found that MBC tumors have specific characteristics, such as a high expression of hormone receptors and low expression of human epidermal growth factor receptor 2 (EGFR2/HER2) [3–7]. In previous studies, MBC has been categorized into type A tumor, type B tumor or type AB tumor [1,8,9]. Type B tumor and neuroendocrine tumor seemed to constitute a spectrum of lesions [10]. Micro-papillary MBC is also considered a special type B subgroup with poor prognosis [11]. Additionally, the microsatellite instability (MSI) phenotype is remarkably rare in MBC compared with mucinous carcinomas present at other anatomical sites [12,13].

Previous clinical studies reported that patients with MBC had bias that resulted from a small sample size or limited time of follow-up due to its relative rarity. The treatment guidelines for optimal local and systemic control of MBC are mostly extrapolated from the treatment experience with IDC and have not undergone rigorous validation in MBC patients. The lack of a particular prognosis evaluation system for MBC has resulted in uniform treatment for MBC.

Currently, nomograms have been developed in the majority of cancer types [14–16]. Nomograms have been accepted as a reliable and alternative tool, or even as a new standard[17], to assist clinicians with making convenient individual predictions. In this study, using a large, nationwide, population-based data, we retrospectively investigated the clinicopathological characteristics of MBC. Furthermore, we attempt to establish a nomogram for patients with MBC based on the clinicopathological data.

## Materials and Methods

### Patient selection and data processing

The Surveillance, Epidemiology, and End Results (SEER) database (http://seer.cancer.gov/) is sponsored by the National Cancer Institution with the aim of collecting information about the cancer incidence and outcome. The current SEER database collects and publishes cancer data from 18 population-based cancer registries among 14 states across the United States, representing approximately 30% of the United States population. The SEER database is collected and released annually, reflecting the most updated information. The SEER data do not capture information about surgery or radiation provided in the past four months of diagnosis nor is there information about recurrence or metastasis that is detected subsequent to the initial diagnosis. We received permission to access the research data (Reference Number: 10263-Nov2015). The study was approved by the review board of Zhejiang University Jinhua hospital. SEER.Stat software was utilized to identify patients with breast cancer in 1990–2007. The specific inclusion criteria were as follows: (1) Years of diagnosis were from 1990 to 2007. (2) Patients without distant metastases. (3) Histological type ICD-O-3 was limited to 8500/3 (IDC) and 8480/3 (MBC). The exclusion criteria were as follows: (1) Patients lacking documentation of race, age at diagnosis and marital status. (2) Patients younger than 20 years old or older than 80 years old. (3) Patients with multiple primary tumors (excluded to make the analyses of cancer-specific survival (CSS) more consistent). (4) Patients who survived less than one month (for the detailed inclusion and exclusion criteria, see **S1 Fig**).

### Statistical analyses

All of the cases were regrouped according to the 7th American Joint Committee on Cancer (AJCC) TNM staging system. Race was divided into white, black and other. The hormone-receptor status of the tumor was stratified to hormone receptor (HoR) -positive [estrogen receptor (ER)-positive/progesterone receptor (PR)-positive, ER-negative/PR-positive and ER-positive/PR-negative] and HoR-negative (ER-negative/PR-negative). The cutoff age of 70 was achieved through the X-tile program [18] **(S1 Fig)**. Age was classified into young (< or = 70 years old) and old (> 70 years old) groups. Marital status was regrouped as married, single (never married or having a domestic partner) or divorce (separated, divorced and widowed).

The distribution of the histological type in different subgroups was analyzed using Chi-Squared tests. The CSS was calculated from the date of diagnosis to the date of death from breast cancer. Survival curves were generated using Kaplan-Meier methods, and the log-rank test was performed to evaluate the survival differences between groups. The 5- and 10-year CSS rates were calculated using the Life-Table method. Multivariable analyses were performed with Cox regression models and adjusted hazard ratios (HRs) along with 95% confidence intervals (CIs) to adjust for prognostic variables. In the multivariable analysis, the T- and N-classification and ER/PR status variables, rather than the stage and HoR status variables, were included to avoid multicollinearity.

A nomogram was constructed based on the results of the Cox proportional hazard model and by using the *rms* package in R software (http://www.r-project.org/). For inclusion into the final nomogram, the effect of the continuous variable, age, was explored using restricted cubic splines with five knots, resulting in a satisfactory sensitivity. The nomogram was internally validated by bootstrapping with 1000 resamples as quantified by the concordance index (C-index). Calibration curves, which plot the average Kaplan-Meier estimate against the corresponding nomogram for 5- or 10- year CSS, are provided to evaluate the nomogram performance.

The probability of CSS in every variable was predicted as a point by the nomogram. The risk score of CSS was calculated for each patient by totaling the points for every variable. Using two cut-off values from the X-tile program, the cohort was classified as three subgroups: low risk = scoring 0–158, medium risk = scoring 159–205 and high risk = scoring 205–416.

In the MBC cohort, the cumulative incidence of breast cancer special death (BCSD) was calculated based on a competing risk regression model [19]. The BCSD was considered as the failure event and non-BCSD as the competing event. The stacked cumulative incidence function plot was used to describe the actual prognosis of specific causes of death [20].

When the two-sided *P* value was less than 0.05, the difference was considered statistically significant. Analyses were performed using statistical software STATA/SE 12.0 (StataCorp LP, TX, USA) and R software (version 3.0.1).

## Results

### Clinicopathological characteristics

A total of 139611 eligible patients with early breast cancer were included in the study. The medium age of the 136569 (97.82%) patients with IDC was 53 years, and it was 75 years in the 3042 (2.18%) patients with MBC. The detailed clinicopathological characteristics according to the histological types are summarized in **Table 1**. Patients with MBC had a higher percentage than IDL in cases with patients over 70 years old (*P* < 0.001). MBC was more common in women of another race (*P* < 0.001). Furthermore, patients with MBC had less lymph node involvement (89.84% vs. 65.29%, *P* < 0.001), an earlier stage (stage I) (68.54% vs. 49.74%, *P* < 0.001), and well-differentiated lesions (59.57% vs. 17.40%, *P* < 0.001). MBC were associated with a higher frequency of ER-positive status (96.75% vs. 84.52%, *P* < 0.001) as well as a dramatically higher frequency of HoR-positive status (97.14%).

**Table 1:** The characteristics of 139611 patients with resectable breast cancer. ER: Estrogen receptor; HoR: Hormone receptor; PR: prognosis receptor; IDC: Infiltrating duct carcinoma; and MBC: mucinous breast cancer.

Risk Factors	*n* (%)	IDC, *n* (%)	MBC, *n* (%)	*P* *
Total	139611	136569(97.82)	3042(2.18)	
Age				<0.001
Younger	115213	113163(82.86)	2050(67.39)	
Older	24398	23406(17.14)	992(32.61)	
Marital status				
Married	87769	86038(63.00)	1731(56.90)	<0.001
Single	17613	17210(12.60)	403(13.25)	
Divorced	34229	33321(24.40)	908(29.85)	
Race				
White	114544	112092(82.08)	2452(80.60)	<0.001
Black	12886	12656(9.27)	230(7.56)	
Other	12181	11821(8.66)	360(11.83)	
Location				<0.001
Central portion of breast	11403	11051(8.09)	352(11.57)	
Upper-inner quadrant	23561	23054(16.88)	507(16.67)	
Lower-inner quadrant	12292	11844(8.67)	448(14.73)	
Upper-outer quadrant	77704	76391(55.94)	1313(43.16)	
Lower-outer quadrant	14651	14229(10.42)	422(13.87)	
Differentiated grade				
Well	25570	23758(17.40)	1812(59.57)	<0.001
Moderate	57856	56792(41.58)	1064(34.98)	
Poor	56185	56019(41.02)	166(5.46)	
T-classification				
T1	90953	88742(64.98)	2211(72.68)	
T2	41656	40925(29.97)	731(24.03)	
T3	4798	4709(3.45)	89(2.93)	<0.001
T4	2204	2193(1.61)	11(0.36)	
N-classification				
N0	91896	89163(65.29)	2733(89.84)	
N1	33160	32908(24.10)	252(8.28)	
N2	9906	9862(7.22)	44(1.45)	
N3	4649	4636(3.39)	13(0.43)	<0.001
Stage ^a^				
I	70018	67933(49.74)	2085(68.54)	
II	52219	51349(37.60)	870(28.60)	
III	17374	17287(12.66)	87(2.86)	
ER				<0.001
Negative	34915	34816(25.49)	99(3.25)	
Positive	104696	101753(74.51)	2943(96.75)	
PR				<0.001
Negative	48284	47813(35.01)	471(15.48)	
Positive	91327	88756(64.99)	2571(84.52)	
HoR				<0.001
Negative	31916	31829(23.31)	87(2.86)	
Positive	107695	104740(76.69)	2955(97.14)	

* *P* values obtained from the χ2 test. All statistical tests were two-sided.

### Survival analysis

**Fig 1:**
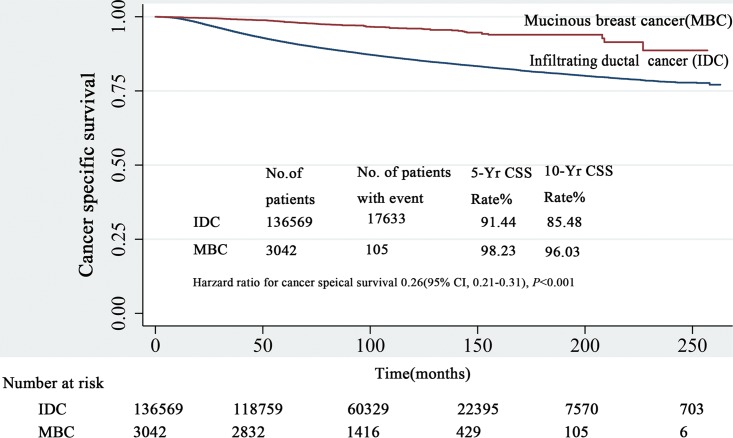
The survival of patients with MBC and IDC by Kaplan-Meier analysis. Patients with MBC obviously had better survival (HR = 0.26; 95% CI, 0.21–0.31, and *P* < 0.001) with 5- and 10-year CSS rates of 98.23% and 96.03% versus 91.44% and 85.48% in patients with IDC, respectively. IDC: infiltrative ductal cancer; MBC: mucinous breast cancer; and CSS: cancer specific survival.

**Fig 2:**
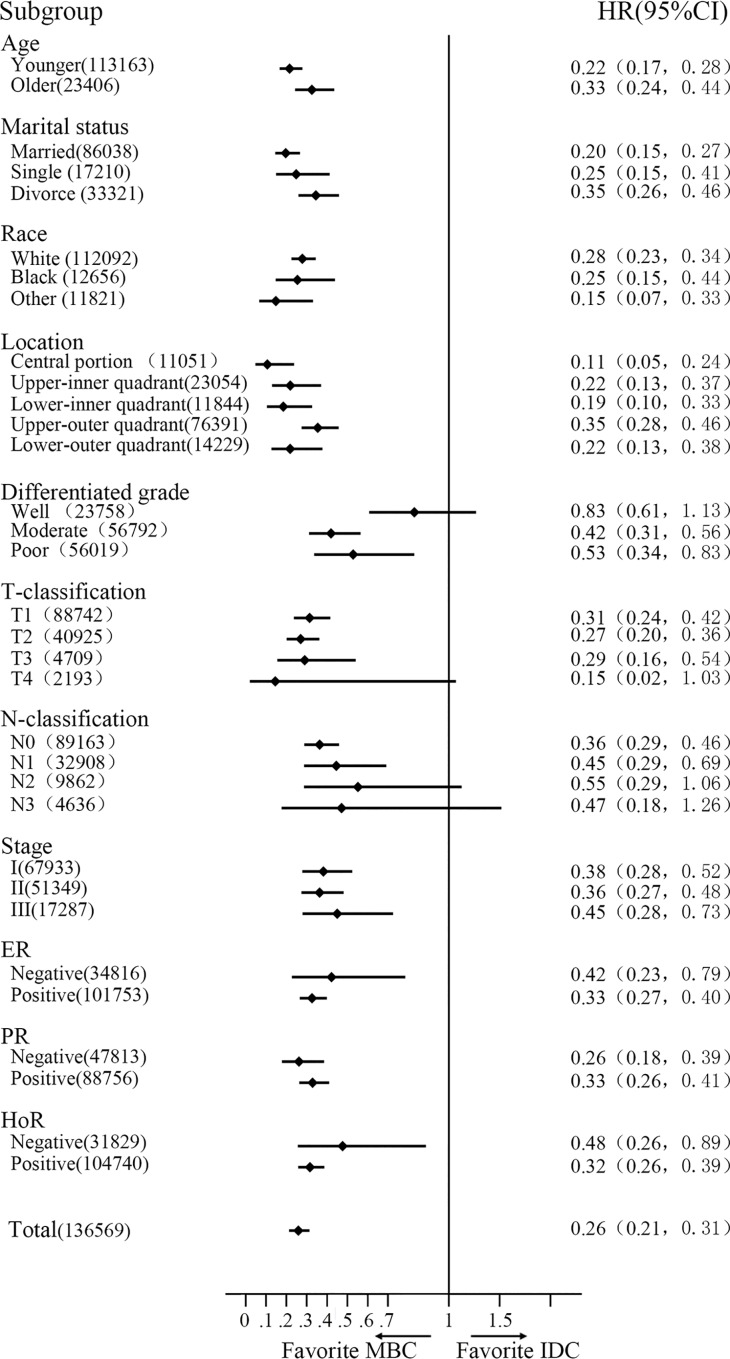
The forest plot of subgroup analysis. Except for the well-differentiated type, T4-classification or N3-classification subgroup, patients with MBC had better outcomes than patients with IDC. IDC: infiltrative ductal cancer; MBC: mucinous breast cancer; HR: hazard risk; and CI: confidence index.

The median follow-up was 91 months (range 1–263 months). Patients with MBC obviously had better survival (HR = 0.26; 95% CI, 0.21–0.31, *P* < 0.001). The 5- and 10-year CSS rates of MBC were 98.23% and 96.03%, respectively, while 91.44 and 85.48% were observed for patients with IDC **(Fig 1)**. Multivariate analysis with the Cox regression model showed that MBC was an independently better prognostic factor (HR = 0.62; 95% CI, 0.51–0.75; *P* < 0.001). Furthermore, we stratified the entire cohort by histological type and analyzed CSS according to patient and tumor characteristics **(Fig 2)**. The forest plot of subgroup analysis revealed that except for the well-differentiated type, T4-classification or N3-classification subgroup, patients with MBC had favorite outcomes compared with patients with IDC.

### Prognostic factors in MBC

**Fig 3:**
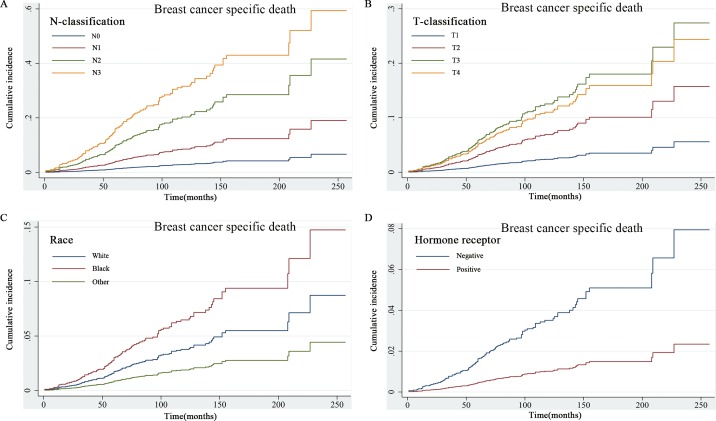
Univariate analysis based on the competing risk regression model. Factors of N-classification (3A), T-classification (3B), Race (3C) and HoR status (3D) were significant prognostic factors for survival.

The competing risk regression model was used to explore the prognostic factors for patients with MBC. Univariate analysis revealed that age, marital status, tumor location, differentiated grade, T and N-classification, and ER/PR status were statistically significant prognostic factors for survival **(Table 2, Fig 3)**. Based on the results of univariate analysis, further multivariate analysis showed that old age, poorly differentiated tumor and advanced T and N-classification were significantly associated with worse prognosis.

**Table 2:** The characteristics of 3042 patients with resectable mucinous breast cancer. ER: Estrogen receptor; HoR: Hormone receptor; and PR: prognosis receptor.

Risk Factors	Univariate analysis *		Multivariate analysis *	
	HR (95% CI)	*P*	HR (95% CI)	*P*
Age				
Younger	-	-	-	-
Older	1.56(1.06–2.3)	0.023	1.97(1.32–2.95)	0.001
Marital status				
Married	1	-	1	-
Single	1.67(0.93–3)	0.089	1.57(0.87–2.85)	0.134
Divorced	2.18(1.44–3.3)	<0.001	1.95(1.26–3)	0.003
Race				
White	1	-	1	-
Black	1.74(0.97–3.13)	0.062	1.18(0.64–2.17)	0.594
Other	0.5(0.22–1.13)	0.097	0.49(0.21–1.13)	0.093
Location				
Central portion of breast	1	-	1	-
Upper-inner quadrant	1.66(0.64–4.32)	0.296	2.16(0.8–5.82)	0.129
Lower-inner quadrant	1.61(0.61–4.29)	0.338	2.26(0.82–6.19)	0.114
Upper-outer quadrant	2.7(1.17–6.23)	0.020	2.92(1.23–6.93)	0.015
Lower-outer quadrant	1.83(0.7–4.81)	0.221	2.41(0.87–6.7)	0.092
Differentiated grade				
Well	1	-	1	-
Moderate	1.72(1.12–2.63)	0.013	1.35(0.87–2.1)	0.185
Poor	4.98(2.89–8.59)	<0.001	2.86(1.51–5.41)	0.001
T-classification				
T1	1	-	1	-
T2	2.98(1.99–4.47)	<0.001	2.07(1.31–3.25)	0.002
T3	5.57(2.81–11.06)	<0.001	3.12(1.34–7.27)	0.008
T4	4.86(0.71–33.33)	0.108	3.16(0.35–28.88)	0.308
N-classification				
N0	1	-	1	-
N1	3.07(1.87–5.04)	<0.001	2.23(1.31–3.79)	0.003
N2	7.81(3.85–15.85)	<0.001	4.1(1.62–10.37)	0.003
N3	13.08(4.72–36.3)	<0.001	6.46(1.91–21.82)	0.003
Stage				
I	1	-	-	-
II	3.2(2.11–4.87)	<0.001	-	-
III	11.38(6.43–20.14)	<0.001	-	-
ER				
Negative	1	-	1	-
Positive	0.33(0.17–0.65)	0.001	0.57(0.24–1.34)	0.197
PR				
Negative	1	-	1	-
Positive	0.57(0.36–0.89)	0.014	0.71(0.42–1.21)	0.210
HoR				
Negative	1	-	-	-
Positive	0.29(0.15–0.56)	<0.001	-	-

* Univariate and multivariate analyses were conducted using the competing risk regression model.

**Fig 4:**
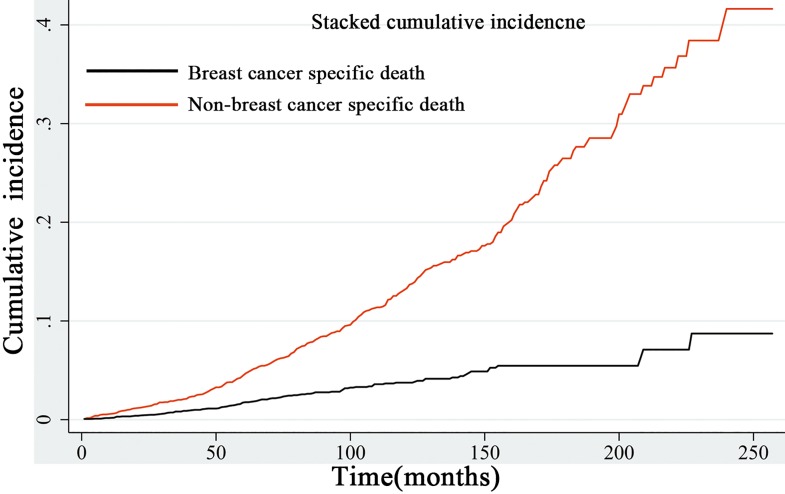
Stacked cumulative incidence plot of breast cancer-specific survival and non-breast cancer-specific survival. Non-breast cancer specific survival had a predominant impact on the survival.

The stacked incidence cumulative plot showed that non-BCSD had a predominant impact on survival. The risk of BCSD was exceeded by non-BCSD in the early course of follow-up, and the trend was increasingly obvious with the passage of time **(Fig 4)**.

### Construction and validation of a prognostic nomogram model in MBC

**Fig 5:**
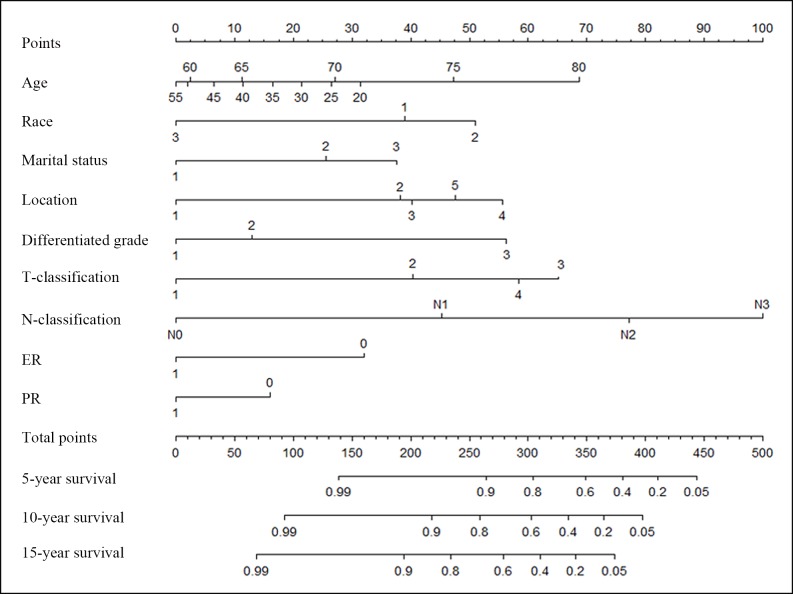
Nomogram for predicting the CSS of patients with MBC. The nomogram is used by summing the points identified on the top scale for each independent covariate. The total points projected to the bottom scale indicate the % probability of the 5-, 10-, and 15-year survival. Race: 1 = white, 2 = black and 3 = other; Marital status: 1 = married; 2 = single (never married or having a domestic partner) and 3 = divorced (separated, divorced, or widowed). Location: 1 = central portion of the breast, 2 = upper-inner quadrant of the breast, 3 = lower-inner quadrant of the breast, 4 = upper-outer quadrant of the breast and 5 = lower-outer quadrant of breast. T and N-classification according to the 7^th^ AJCC TNM system. ER = estrogen receptor: 1 = positive and 0 = negative. PR = progesterone receptor: 1 = positive and 0 = negative. CSS: cancer specific survival and MBC: mucinous breast cancer.

Significant factors identified by the Cox model were used to build a nomogram to predict the probability of CSS in MBC patients **(Fig 5)**. The tumor location, differentiation grade, T and N-classification, and ER and PR statuses were included. Baseline characteristics, such as the age, race and marital status were also incorporated into the model. The nomogram illustrated T and N-classification as the largest contributor to the prognosis, which was followed by the differentiation grade. Each subtype for all variables was assigned a score on the point scale **(Table 3)**. By summing the total score and locating it on the total point scale, we were easily able to draw a straight line down to estimate 5-, 10- or 15- year predicted CSS rate.

**Table 3:** Point assignment and prognostic score in the nomogram. CSS: cancer specific survival.

Variable	Score	Estimated 5-year CSS rate (%)
Age (years)		
20	31	
25	26	
30	21	
35	16	
40	11	
45	6	
50	2	
55	0	
60	2	
65	11	
70	27	
75	47	
80	69	
Marital status		
Married	0	
Single	26	
Divorced	38	
Race		
White	39	
Black	51	
Other	0	
Location		
Central portion of breast	0	
Upper-inner quadrant	38	
Lower-inner quadrant	40	
Upper-outer quadrant	56	
Lower-outer quadrant	48	
Differentiated grade		
Well	0	
Moderate	13	
Poor	56	
T-classification		
T1	0	
T2	40	
T3	65	
T4	58	
N-classification		
N0	0	
N1	45	
N2	77	
N3	100	
ER		
Negative	32	
Positive	0	
PR		
Negative	16	
Positive	0	
Total prognostic score		
> = 444		0.05
410–443		0.2
380–409		0.4
349–379		0.6
305–348		0.8
264–304		0.9
139–263		0.99

**Fig 6:**
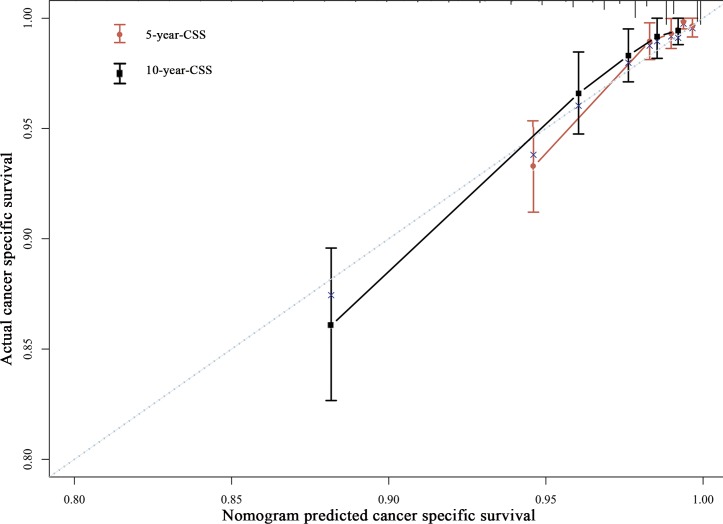
The calibration curve for predicting patient CSS rates at 5 and 10 years. The nomogram-predicted probability of CSS is plotted on the *x*-axis; the actual CSS is plotted on the *y*-axis. CSS: cancer specific survival and MBC: mucinous breast cancer.

The internal validation using the bootstrap method showed the nomogram can accurately predict the CSS with a C-index of 0.816 (95% CI, 0.773–0.859). The calibration plots demonstrated an excellent agreement between the nomogram prediction and actual observation for the 5- and 10- year CSS rates **(Fig 6)**.

The risk score developed from the nomogram as a continuous variable acted as a prognostic factor for CSS (HR = 1.02, *P* < 0.001). Then, the MBC cohort was classified into three subgroups (low risk, score < 158; medium risk, score of 158–205 and high risk, score > 205) according to the risk score by two cutoff values from the X-tile program **(S2 Fig)**. The univariate analysis showed that the Risk score was significantly associated with the prognosis; the 5- and 10-year CSS rates were 99.53% and 98.87% in the low risk subgroup, 87.92% and 94.02% in the medium risk subgroup, and 90.14% and 80.68% in the high risk subgroup, respectively. The C-index of the model based on the Risk score to predict CSS was 0.789, which was statistically higher than the AJCC TNM staging system (C-index = 0.704, *P* < 0.001).

## Discussion

MBC is a rare histological cancer type with lower malignancy and a comparatively better prognosis. A series of previous studies reported that MBC had different characteristics than other histological breast cancer types. However, some studies showed that there was no longer a survival difference between the patients with MBC and IDC after adjusting for the tumor size and lymph node status [3,21]. In our study, patients with MBC had an obviously better prognosis than patients with IDC, even after adjusting for clinicopathological factors.

The features related to the prognosis of patients with MBC remain controversial. The validity of the intrinsic subtype as a prognostic factor is widely accepted by clinicians. The current NCCN guideline recommends using the ER and PR statuses as the most important factors for making clinical decisions. However, in our study, multivariate analysis showed that ER and PR cannot independently predict the MBC prognosis. This is partly because the rate of ER and PR positivity is too high in our study, which will make it difficult to define the prognostic effect of the ER and PR statuses. Additionally, the HoR positivity (97.14%) is dramatically high and the remaining 3% of patients who are HoR negative are difficult to further stratify. It is not practicable to guide clinical practice according to the ER or PR status.

The gene signature is widely used to predict the prognosis of patents with ER positive and lymph node negative status (most MBC patients have such characteristics) [22,23]. However, for MBC tumor, abundant mucinous content in the tumor will impact the RNA quality of RNA. As a result, the 21-gene assay and MammaPrint based on real-time PCR will no longer be suitable for testing [24].

In previous studies, lymph node involvement was recognized as the most important prognostic factor [1,25,26]. In the current study, the effect of lymph node involvement was predominate in the nomogram. For MBC patients with lymph node involvement (10%), only 1.45% of patients had 1–3 lymph node metastases, and 0.43% of patients had more than three lymph node metastases. Therefore, sentinel lymph node biopsy may be sufficient to evaluate axillary lymph node metastasis.

The prognostic significance of the tumor size is an interesting but controversial issue in MBC patients. In the past, the NCCN guidelines recommended that patients with a tumor larger than 2 cm should receive adjuvant chemotherapy. However, the guidelines have been modified; now, lymph node involvement alone is considered as an indication for chemotherapy, regardless of the T-classification. Although the tumor size is associated with a delay in diagnosis in tumors with low invasion, the prognostic significance of the tumor size is questioned because the production of abundant extracellular mucin is included in the size measurement. As a result, the measured tumor size might fail to reflect the actual tumor size. This complicates the predictive role of the tumor size for MBC. One small size sample study showed that the MBC is associated with a larger tumor compared with IDC [27]. Additionally, one study showed that lymph node involvement is not associated with the tumor size [28]. In our study, the nomogram showed that T3 and T4 tumors had a worse prognosis than T1 and T2 tumors. Therefore, tumor size larger than 5 cm could be considered a poor prognostic factor.

Additionally, a poor differentiation grade is a poorer prognostic factor in the current study. The subgroup showed that MBC had a similar prognosis as IDC in the well-differentiated tumor subgroup, which may improve the prognosis for well-differentiated IDC tumor.

Two published studies in which the MBC prognosis was analyzed using a SEER dataset revealed that patients with MBC were more likely to be older women, had less lymph node involvement and had a better prognosis than IDC patients[5,29]. These findings are consistent with our study. In their studies, a considerable number of patients were diagnosed before 1990 without details about the ER and PR statuses or T- and N-classification. In the current study, detailed information on the T- and N- classification and ER or PR status are presented. Subgroup analysis with these factors was also performed to identify the impact of MBC on survival. Furthermore, we constructed a nomogram to individually predict the CSS. This could more directly help clinicians determine the probability of specific death for individual patients. To the best of our knowledge, this is the first large-population study to construct a nomogram for patients with early MBC.

A stacked cumulative incidence plot surprisingly showed that non-BCSD was predominant events of death. In the previous studies, non-BCSD was never considered in the survival analysis. Based on the results, we further proposed that for patients with early MBC, a competing risk regression model (non-BCSD as competing events) is more rational for analyzing the survival outcome. De Glas also recommended using a competing risk regression model rather than a Cox proportional hazard model that would otherwise overestimate the absolute risk of death in studies of mainly older patients with HoR+ breast cancer [30]. To the best of our knowledge, we first analyzed the prognosis of MBC using a competing risk model.

Our study has several potential limitations. First, we failed to differentiate between MBC subtypes, such as types A, B and AB. Then, the details about other prognostic factors, such the HER2 status and Ki67, as well as information about adjuvant therapy are lacking. Furthermore, external validation of the nomogram was not performed in our study. Despite these limitations, our study shed new light on the impact of MBC on the prognosis of breast cancer patients. Currently, there are no effective tools for predicting the prognosis of patients with MBC. Our study is the first to develop a clinical nomogram that could help clinicians in daily practice. More importantly, the competing risk regression model is recommended as a substitute for the traditional Cox model to decrease the bias of non-BCSD in the MBC survival analysis.

## Supporting Information

S1 Fig The optimal cut-off value for age. **(A)** The optimal cut-off value highlighted by the black circle in the rectangular X-tile plot. **(B)** The histogram of the entire cohort. **(C)** The Kaplan-Meier plot: The cancer-specific survival (CSS) curve of young, older and oldest patients. The young and older groups have similar survival. The age of 70 is chosen as the optimal cut-off value. **(D)** The relative risks (RRs) for all cut-off values from low to high (left to right, x-axis). The RRs are calculated as: events in the older group / event risk in the younger group.
(TIF)

S2 Fig The cut-off point of the risk score. **(A)** The optimal cut-off value is highlighted by the black circle in the triangular X-tile plot. (low risk group, score<158; medium risk group, score of 158–205 and high risk group, score >205). **(2)** The histogram of the entire cohort. **(C)** The Kaplan-Meier plot: The cancer-specific survival curve of younger and older group have similar survival. The age of 70 is chosen as the optimal cut-off value. **(D)** The relative risks (RRs) for all cut-off values from low to high (left to right, x-axis). RRs are calculated as the events in the older group / event risk in the younger group.
(TIF)

## Abbreviations

MBC: mucinous breast cancer
IDC: infiltrative ductal breast cancer
HoR: hormone receptor
ER: estrogen receptor
PR: progesterone receptor
CSS: cancer specific survival
BCSD: breast cancer special death
IQR: inter quartile range
HRs: hazard ratios
CIs: confidence intervals
AJCC: American Joint Committee on Cancer
SEER: Surveillance, Epidemiology, and End Results
BCSD: Breast cancer special death
NCCN: National Comprehensive Cancer Network
