# Supplementary figures and images for: Clinical Nomogram for Predicting Survival Outcomes in Early Mucinous Breast Cancer
Source: PLoS One. 2016 Oct 19;11(10):e0164921. doi: 10.1371/journal.pone.0164921 (PMC5070827; doi:10.1371/journal.pone.0164921)

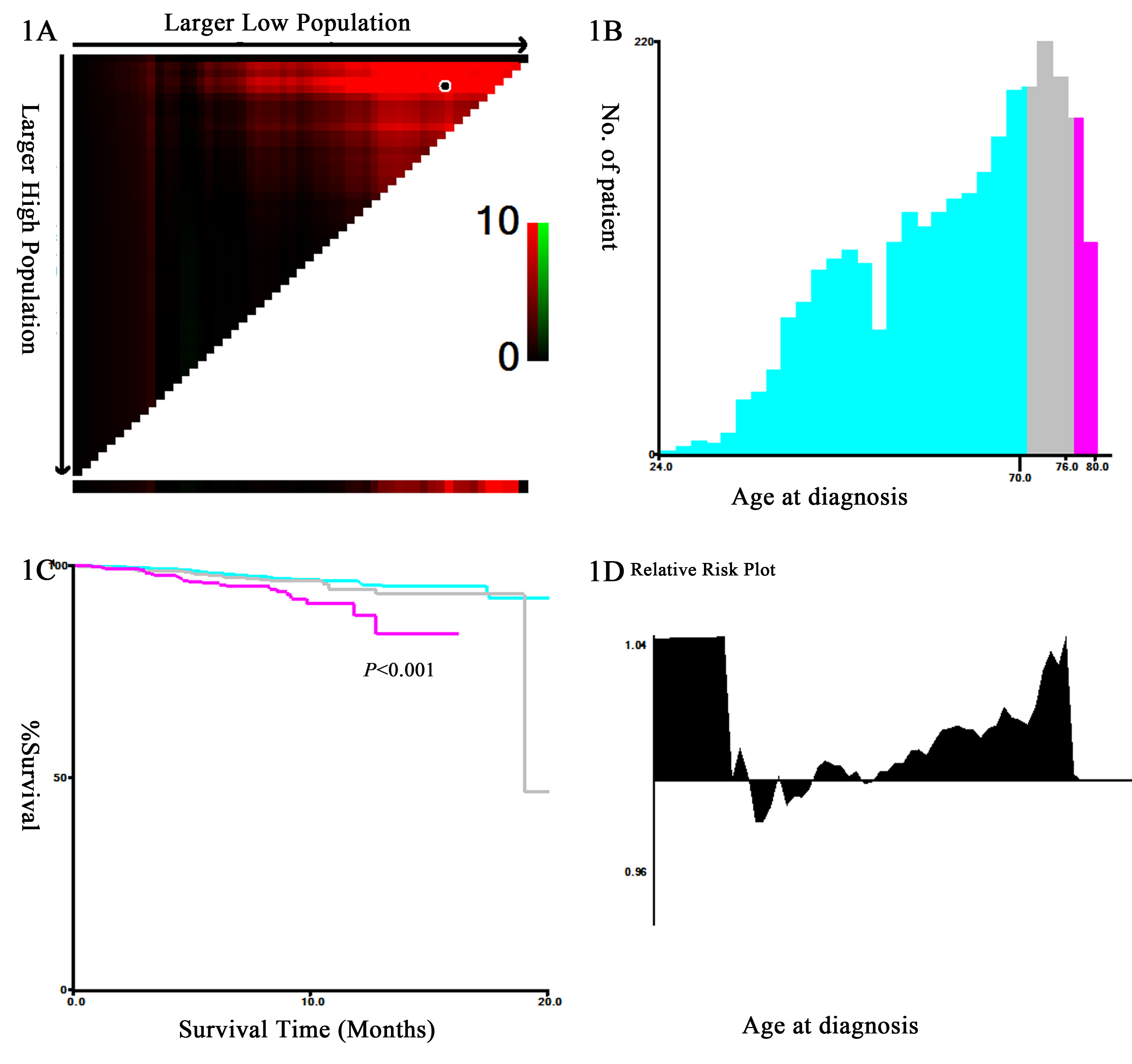

Supplement: S1 Fig — (A) The optimal cut-off value highlighted by the black circle in the rectangular X-tile plot. (B) The histogram of the entire cohort. (C) The Kaplan-Meier plot: The cancer-specific survival (CSS) curve of young, older and oldest patients. The young and older groups have similar survival. The age of 70 is chosen as the optimal cut-off value. (D) The relative risks (RRs) for all cut-off values from low to high (left to right, x-axis). The RRs are calculated as: events in the older group / event risk in the younger group. (TIF) [file pone.0164921.s001.tif]

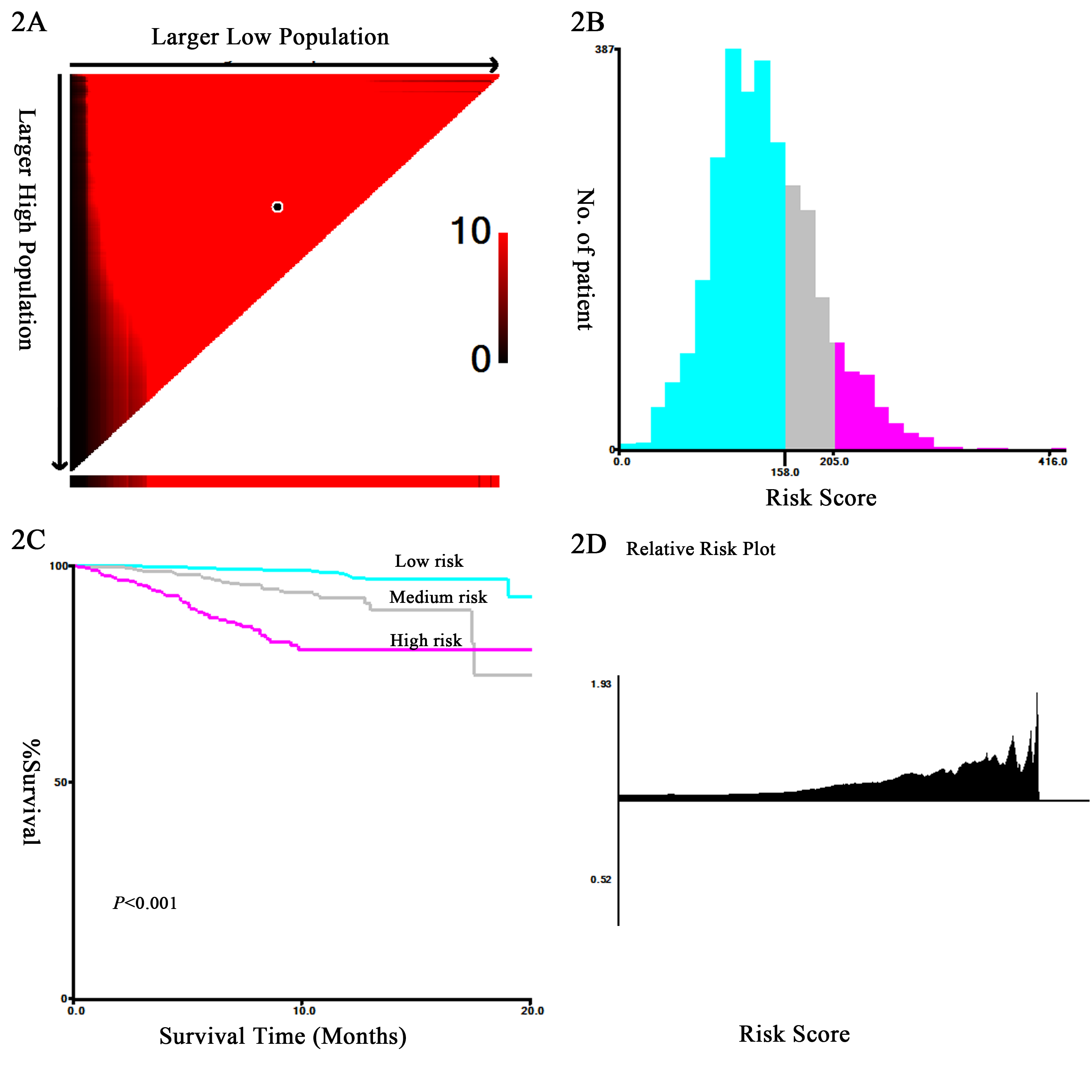

Supplement: S2 Fig — (A) The optimal cut-off value is highlighted by the black circle in the triangular X-tile plot. (low risk group, score<158; medium risk group, score of 158–205 and high risk group, score >205). (2) The histogram of the entire cohort. (C) The Kaplan-Meier plot: The cancer-specific survival curve of younger and older group have similar survival. The age of 70 is chosen as the optimal cut-off value. (D) The relative risks (RRs) for all cut-off values from low to high (left to right, x-axis). RRs are calculated as the events in the older group / event risk in the younger group. (TIF) [file pone.0164921.s002.tif]
